# Coinheritance of germline mutations in *APC* and *MUTYH* genes defines the clinical outcome of adenomatous polyposis syndromes

**DOI:** 10.1016/j.gendis.2022.11.017

**Published:** 2022-12-27

**Authors:** Giovanna Forte, Filomena Cariola, Antonia Lucia Buonadonna, Anna Filomena Guglielmi, Andrea Manghisi, Katia De Marco, Valentina Grossi, Candida Fasano, Martina Lepore Signorile, Paola Sanese, Rosanna Bagnulo, Nicoletta Resta, Vittoria Disciglio, Cristiano Simone

**Affiliations:** a Medical Genetics, National Institute of Gastroenterology “S. de Bellis” Research Hospital, Castellana Grotte, Bari 70013, Italy; b Medical Genetics, Department of Precision and Regenerative Medicine and Jonic Area (DiMePRe-J), University of Bari Aldo Moro, Bari 70124, Italy

Familial adenomatous polyposis (FAP) and *MUTYH*-associated polyposis (MAP) are colon cancer predisposition syndromes. FAP is an autosomal dominant inherited condition caused by germline mutations in the adenomatous polyposis coli (APC) gene and characterized by hundreds to thousands of colorectal adenomas. FAP can be classified into several clinical forms, including profuse FAP (>1,000 adenomas), intermediate FAP (100–1,000 adenomas), attenuated FAP (AFAP) (<100 adenomas), and gastric polyposis and desmoid FAP (GD-FAP) (<50 adenomas). FAP patients also have an increased risk of extra-colonic manifestations.^1^ MAP is inherited in an autosomal recessive manner. Monoallelic *MUTYH* mutation carriers are at increased risk for colonic and extra-colonic cancer. Biallelic *MUTYH* mutations are associated with colorectal polyposis and an increased lifetime risk of gastrointestinal cancers.^2^

Here we report the clinical and family cancer history of two siblings exhibiting colonic polyposis and harboring concurrent germline pathogenic variants in *APC* (c.1111G>T, p.Gly371∗) and *MUTYH* (c.536A>G, p.Tyr179Cys). We compared their clinical phenotype with that of other affected family members and patients described in literature carrying the *APC* c.1111G>T and/or the monoallelic *MUTYH* c.536G>A pathogenic variants. This analysis revealed that patients harboring both variants manifest the disease phenotype earlier than patients carrying only one of them.

In the current study, we examined an Italian family with a clinical diagnosis of adenomatous polyposis syndromes. The index patient was a 45-year-old woman (Fig. S1 II:11) with a family history of colonic polyposis and cancer, which was referred to our Institute for genetic counseling (Supplementary Data).

The index patient (Fig. S1 II:11) was subjected to mutational analysis of the entire *APC* coding region by Sanger sequencing (Supplementary Data), which identified a heterozygous c.1111G>T pathogenic variant in exon 9 (Fig. S2 A). Based on the American College of Medical Genetics and Genomics (ACMG) and the Association for Molecular Pathology (AMP) variant classification scheme, the germline *APC* c.1111G>T substitution can be classified as a pathogenic variant (Supplementary Data). This variant is listed in a major disease-associated database (HGMD Professional), which causes premature protein truncation at codon 371 (p.Gly371∗) and seems to be rare since it has been reported only once in a family with attenuated FAP.^3^ Truncated APC proteins lose their ability to regulate β-catenin degradation, which leads to nuclear accumulation of β-catenin and activation of the Wnt signaling pathway, thereby contributing to polyp formation.^1^ The germline *APC* c.1111G>T pathogenic variant was also detected in the other affected first-degree relatives tested, *i.e.*, the mother (Fig. S1 I:8), a sister, and the brother (Fig. S1 II: 12, II:13), but not in the two unaffected sisters (Fig. S1 II:14, II:15). Surprisingly, another sister (Fig. S1 II:10) with the adenomatous polyposis phenotype did not carry this pathogenic variant. Based on the paternal family history, we subjected her genomic DNA to NGS genetic testing using a panel of 25 hereditary colon cancer-related genes (Supplementary Data). This analysis identified a monoallelic c.536A>G (p.Tyr179Cys) substitution in the *MUTYH* gene, which was confirmed by Sanger sequencing (Fig. S2B, C). Based on the ACMG/AMP variant classification scheme, the germline *MUTYH* c.536A>G substitution can be classified as a pathogenic variant (Supplementary Data). The *MUTYH* c.536A>G germline pathogenic variant was also identified in the index patient's father (Fig. S1 I:7), who was wild-type for *APC*, and in the index patient's *APC*-mutated sister and brother (Fig. S1 II:12, II:13), who therefore carried concurrent *APC* and *MUTYH* pathogenic variants. *MUTYH* encodes a DNA glycosylase able to recognize and correct G:C to T:A transversions resulting from DNA replication or recombination. Notably, c.536A>G (p.Tyr179Cys) is the most prevalent *MUTYH* pathogenic variant in Caucasians, together with c.1187G>A.^2^ Functional assays have shown that the *MUTYH* c.536A>G substitution reduces its glycosylase activity by 98%.^2^ Moreover, homozygous *MUTYH* c.536A>G pathogenic variants are associated with a more severe MAP clinical course than other homozygous *MUTYH* mutations and a higher risk of developing CRC than homozygous c.1187G>A or compound heterozygous c.536A>G/c.1187G>A *MUTYH* variants.^2^ Monoallelic *MUTYH* mutations occur only in 1%–2% of the Caucasian population.^2^ Various studies have shown that monoallelic *MUTYH* mutation carriers have an approximately 2.5-fold increased risk of CRC, an elevated risk of liver and gastric cancer, and a slightly increased risk of breast cancer.^2^ Moreover, several studies reported an increased risk of CRC in monoallelic *MUTYH* c.536A>G carriers compared to monoallelic *MUTYH* c.1187G>A carriers.^4^

This is consistent with the cancer history of the family examined in the present study. Indeed, the index patient's father (Fig. S1 I:7) developed CRC at 66 years of age and a paternal aunt (Fig. S1 I:6) was diagnosed with GC at 40 years of age. Concurrent germline mutations in two different genes associated with colon cancer predisposition syndromes, like *APC* and *MUTYH*, are rare events that may influence disease progression. Previously, Li et al described four subjects with concurrent *APC* and *MUTYH* mutations different from those detected in this study. Furthermore, the authors reported that disease onset in patients with concurrent *APC* and *MUTYH* mutations occurred earlier than in patients carrying only *MUTYH* mutations and similar to patients carrying only *APC* mutations.^5^ However, this comparison was made regardless of the type of *APC* or *MUTYH* alterations.

Phenotypic analysis of our index patient's family members revealed that the median age of diagnosis was slightly lower for patients concurrently harboring the *APC* c.1111G>T and the monoallelic *MUTYH* c.536A>G pathogenic variants (median 42.5 years, range 42–43) compared to patients carrying only the *APC* (median 53 years, range 43–63) or the *MUTYH* (median 46 years, range 40–55) pathogenic variant (Table 1A). The earlier manifestation of the disease phenotype in patients harboring concurrent *APC* c.1111G>T and *MUTYH* c.536A>G pathogenic variants suggests that they may have an additive effect. Based on this finding, we performed a literature review of the *APC* c.1111G>T and the monoallelic *MUTYH* c.536A>G pathogenic variants reported in HGMD Professional to ascertain whether this trend was confirmed in a larger population. This analysis identified only 37 subjects, including those examined in this paper, with colonic polypoid lesions and/or CRC. Of these, 7 patients carried the *APC* c.1111G>T pathogenic variant, 28 patients carried the *MUTYH* c.536A>G pathogenic variant, and 2 patients concurrently harbored both (Table S1)*.* A comparison of their clinical data corroborated our observation. Indeed, the median age of diagnosis of colorectal polypoid lesions and/or CRC was 50 years (range 36–63) for the *APC* c.1111G>T pathogenic variant and 61 years (range 40–79) for the *MUTYH* c.536A>G pathogenic variant, while an earlier disease diagnosis (42.5 years, range 42–43) trend was observed in patients carrying both (Table 1B).

**Table 1A:** Analysis of family members' clinical data.

Genotype		*n*	Median age of diagnosis (y)	Range
APC	MUTYH			
*WT*	*C.536A>G*	3	46	40–55
*c.1111G>T*	*C.536A>G*	2	42.5	42–43
*c.1111G>T*	*WT*	4	53	43–63

WT = wild-type; y = years.

**Table 1B:** Analysis of clinical data from literature review.

Genotype		*n*	Median age of diagnosis (y)	Range
APC	MUTYH			
*WT*	*C.536A>G*	28	61	40–79
*c.1111G>T*	*C.536A>G*	2	42.5	42–43
*c.1111G>T*	*WT*	7	50	36–63

WT = wild-type; y = years.

A possible explanation of the more severe clinical phenotype detected in patients concurrently harboring both genetic alterations is that monoallelic *MUTYH* c.536A>G pathogenic variants, which reduce the glycosylase activity of the protein, can lead to the early accumulation of errors in cancer driver genes. These unrepaired DNA errors may represent the additional hit responsible for adenomatous polyposis syndromes in subjects already carrying a pathogenic *APC* variant. Moreover, the earlier occurrence of colorectal adenomas may foster disease progression and accelerate CRC development. We are aware that the main limitation of our study is the low number of *APC* c.1111G>T/*MUTYH* c.536A>G carriers. This is mainly due to the fact that patients carrying concurrent germline mutations in two important genes associated with colon polypoid lesions and/or gastrointestinal cancer are very rare. Further studies investigating the mutational status and clinical manifestations of a larger number of patients with colonic polypoid lesions and/or gastrointestinal cancer are needed to confirm the additive effect of the *APC* c.1111G>T and *MUTYH* c.536A>G pathogenic variants and their contribution to the clinical phenotype of patients with adenomatous polyposis syndromes. This study emphasizes the importance of evaluating detailed family history and testing multiple gene panels in patients with hereditary cancer to provide tailored genetic counseling, management, and surveillance to families with adenomatous polyposis syndromes.

## Conflict of interests

The authors have no competing interests to declare.

## Funding

The research leading to these results has received funding from AIRC under IG 2019—ID. 23794 project—to Cristiano Simone. Furthermore, this work was funded by the research funding program “Ricerca Corrente 2019–2021; 2021-2023” to Cristiano Simone, “Ricerca Corrente 2022-2024” to Vittoria Disciglio, “Ricerca Corrente 2022-2024” to Candida Fasano, AIRC fellowship for Italy “ID. 26678-2021” to Martina Lepore Signorile, “Starting Grant” SG-2019-12371540 to Paola Sanese from the Italian Ministry of Health and the 2017 PRIN (Research Projects of National Relevance) n. 2017WNKSLr-LS4 from the Italian MIUR to Cristiano Simone.
