# Supplementary figures and images for: Coinheritance of germline mutations in APC and MUTYH genes defines the clinical outcome of adenomatous polyposis syndromes
Source: Genes Dis. 2022 Dec 27;10(4):1187–9. doi: 10.1016/j.gendis.2022.11.017 (PMC10311103; doi:10.1016/j.gendis.2022.11.017)

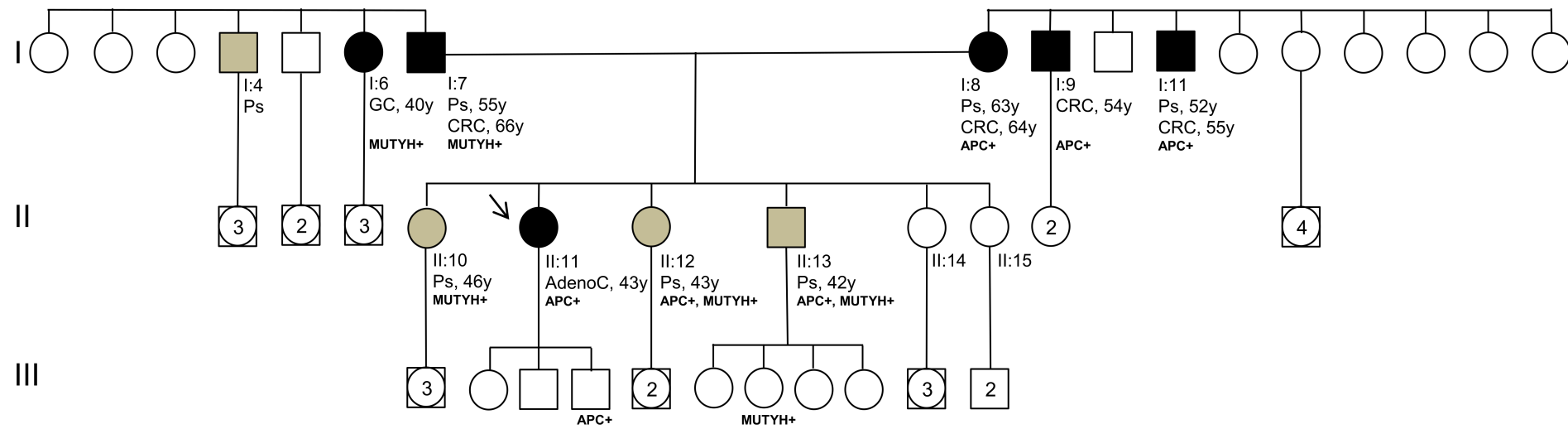

Supplement: Multimedia component 1 [file mmc1.pdf]

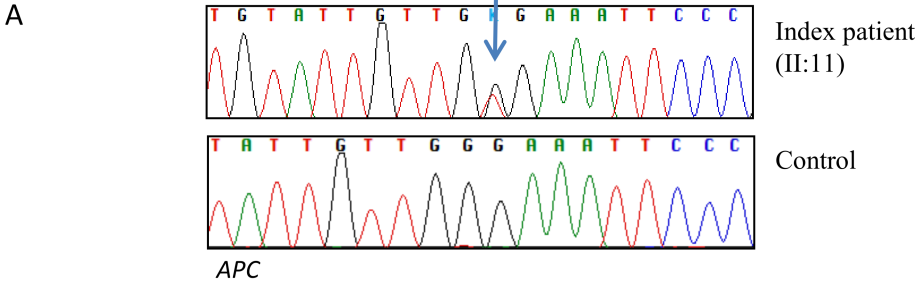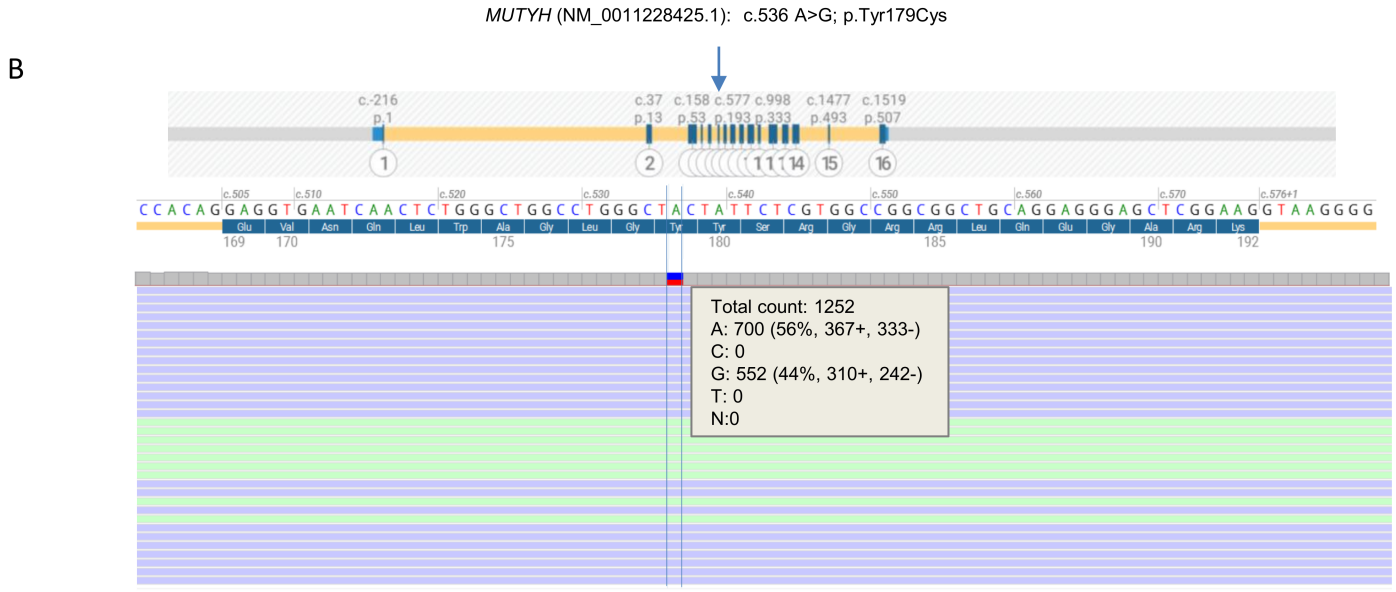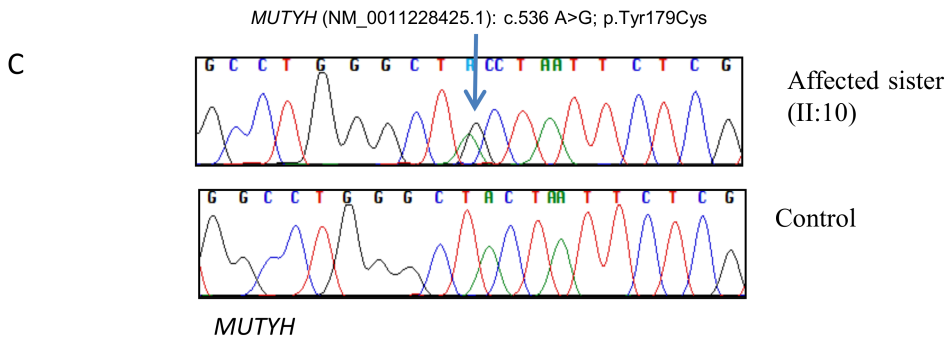

Supplement: Multimedia component 2 [file mmc2.pdf]
