# Supplementary material for: Coinheritance of germline mutations in APC and MUTYH genes defines the clinical outcome of adenomatous polyposis syndromes
Source: Genes Dis. 2022 Dec 27;10(4):1187–9. doi: 10.1016/j.gendis.2022.11.017 (PMC10311103; doi:10.1016/j.gendis.2022.11.017)
Supplement: Multimedia component 3 [file mmc3.docx]

**Supplementary Figures Legend**

Supplementary Figure 1

Pedigree of the family involved in this study. Squares indicate men, circles represent women. Squares and circles with a number inside represent multiple individuals. The arrow indicates the index case. Black-filled symbols denote individuals with cancer, grey-filled symbols correspond to patients with gastrointestinal polyps and unfilled symbols indicate unaffected individuals. The following information is given below each filled symbol: clinical manifestations (CRC = colorectal cancer; GC = gastric cancer; Ps = gastrointestinal polyps; AdenoC = colorectal adenoma), age at diagnosis (y = years), mutational status of *APC* and *MUTYH* genes.

Supplentary Figure 2

A) Sequencing electropherograms of genomic DNA from the index patient (II:11), showing the c.1111G>T substitution in the *APC* gene.

B) Next-generation sequencing results showing the *MUTYH* c.536A>G substitution in genomic DNA from an affected sister (II:10) of the index patient. Visualization of the BAM (Binary Alignment Map) file of the variant using Alamut Visual Plus software v.1.6.1 (Interactive Biosoftware, Rouen, France). A partial sequence of *MUTYH* (NM_001128425.1) is shown. The position of the c.536A>G (p.Y179C) mutation in *MUTYH* exon 7 is highlighted by the blue arrow and line. The frequency and absolute number of reads are indicated for the mutant and wild-type alleles.

C) Sequencing electropherograms of genomic DNA from an affected sister (II:10) of the index patient, confirming the missense *MUTYH* mutation detected in B.
