# Supplementary material for: Coinheritance of germline mutations in APC and MUTYH genes defines the clinical outcome of adenomatous polyposis syndromes
Source: Genes Dis. 2022 Dec 27;10(4):1187–9. doi: 10.1016/j.gendis.2022.11.017 (PMC10311103; doi:10.1016/j.gendis.2022.11.017)
Supplement: Multimedia component 4 [file mmc4.docx]

# Family History

The index patient was a 45-year-old woman (Fig. S1 II:11) with a family history of colonic polyposis and cancer, which was referred to our Institute for genetic counseling.

She underwent total colectomy and ileorectal anastomosis at 43 years of age, which revealed the presence of more than 100 adenomatous polypoid and non-polypoid lesions. Histological examination of the lesions showed sessile and pedunculated tubular and tubulovillous adenomas with moderate-grade dysplasia in the large intestine. Moreover, one lesion exhibited high-grade dysplasia. One sister (Fig. S1 II:10) developed a tubular adenoma with low-grade dysplasia at 46 years of age and a 0,5 cm diameter sessile polyp at 47 years of age. Another sister (Fig. S1 II:12) and the brother (Fig. S1 II:13) exhibited several adenomatous polypoid lesions with moderate-grade dysplasia in the colon at 43 and 42 years of age, respectively. The mother (Fig. S1 1 I:8) developed stenosing cancer in the rectum and sigmoid colon at 64 years of age. In addition, two maternal uncles (Fig. S1 I:9; I:11) were diagnosed with CRC at 54 and 55 years of age, respectively. The father (Fig. S1 I:7) underwent right hemicolectomy for CRC at 66 years of age. A paternal aunt (Fig. S1 I:6) developed gastric cancer (GC) at 40 years of age, and a paternal uncle (Fig. S1 I:4) exhibited colon polyps.

# Supplementary Methods

Patient recruitment

Informed consent for genetic analysis was obtained from all subjects analyzed, using a form approved by the competent ethics committee. This study was conducted in full accordance with the principles of the Declaration of Helsinki and any other applicable local ethical and legal requirements (protocol code n. 170, date of approval: October 31, 2016). The molecular testing carried out in this study is based on the routine clinical diagnostic assessment performed at our Institute.

Genetic analysis

DNA extraction and Sanger sequencing

DNA was extracted from peripheral blood cells (PBCs) using the QIAamp Mini Kit (Qiagen, Hilden, Germany) according to the manufacturer’s instructions. The complete coding region of the *APC* (NM_000038.5) and *MUTYH* (NM_001128425.1) genes was screened for mutations as previously described^1–3^. PCR sequencing and capillary electrophoresis were performed on an Applied Biosystems 3130 Genetic Analyzer (Thermo Fisher Scientific, Waltham, MA, USA). Mutations and polymorphisms were confirmed in independently amplified PCR products. The global population frequency of the identified *APC* and *MUTYH* variants was retrieved from the 1000 Genome^4^, gnomAD^5^, dbSNP^6^, and NHLBI Exome Sequencing Project^7^ databases. Inadequately covered regions of the *APC* and *MUTYH* genes and any variants detected through next-generation sequencing analysis were subjected to Sanger sequencing using the BigDye Terminator v1.1 Cycle Sequencing Kit (Thermo Fisher Scientific, Waltham, MA, USA) according to the manufacturer’s instructions on an ABI 310 Genetic Analyzer (Thermo Fisher Scientific, Waltham, MA, USA).

Next-generation sequencing (NGS)

The entire coding region of 25 hereditary cancer-related genes that have a central role in the DNA repair and mismatch repair pathways (*APC, ATM, BARD1, BMPR1A, BRIP1, CDH1, CDK4, CDKN2A, CHEK2, EPCAM, MLH1, MRE11A, MSH2, MSH6, MUTYH, NBN, PALB2, PMS2, PTEN, RAD50, RAD51C, RAD51D, SMAD4, STK11,* and *TP53*) was sequenced using a commercial Ion AmpliSeq™ BRCA Reflex - Hereditary Cancer Research Panel (Life Technologies, Carlsbad, CA, USA). The panel was composed of 610 amplicons divided into 2 primer pools for a total of 74,058 kb of DNA, covering 98.12% of the regions of interest (ROI). The panel library was generated from 10 ng of DNA using the Ion AmpliSeq Library Kit v2.0 (Life Technologies, Carlsbad, CA, USA) according to the manufacturer’s instructions and quantified using the Qubit dsDNA HS Assay Kit (Life Technologies, Carlsbad, CA, USA) on a Qubit2.0 Fluorometer (Life Technologies, Carlsbad, CA, USA). One of 16 barcodes of the Ion Xpress Barcode Adapters 1–16 Kit (Life Technologies, Carlsbad, CA, USA) was added to each sample. Equimolar amounts of each library were used to prepare templates for clonal amplification. Emulsion PCR was performed on a OneTouch2 system (Life Technologies, Carlsbad, CA, USA) using the Ion PGM Template OT2 200 Kit (Life Technologies, Carlsbad, CA, USA). Templates were enriched using Ion OneTouch ES (Life Technologies, Carlsbad, CA, USA) and prepared for loading on a 316v2/318v2 chip. Sequencing runs were performed on an Ion Torrent Personal Genome Machine (Life Technologies, Carlsbad, CA, USA) using the Ion PGM Sequencing 200 Kit v2 (Life Technologies, Carlsbad, CA, USA) according to the manufacturer’s instructions. Data analysis was performed with Torrent Suite Software v.5.0. (Life Technologies, Carlsbad, CA, USA). Reads were aligned to the hg19 human reference genome from the UCSC (University of California Santa Cruz) Genome Browser and to BED files designed using Ion AmpliSeq Designer. Alignments were verified with Alamut® Visual Plus (software v.1.6.1; Interactive Biosoftware, Rouen, France). The mean average read depth and the percentage of reads on target mapping to the ROI were calculated using the Coverage Analysis plugin of Torrent Suite 5.0.4 software. For each sample, the ratio of ROI with a minimum coverage of 200× was determined using the amplicon coverage matrix file. Germline variants in the targeted regions were detected with the Variant Caller plug-in of Torrent Suite 5.0.4 software. The Variant Caller was run with default germline and low stringency settings. Variants were filtered with a minimum of 30× coverage and a sample variant frequency between 30% and 70%. Alamut® Visual was also used for variant interpretation to eliminate strand bias and reduce false positive calls. Common single nucleotide variants (minor allele frequency [MAF] >5%), exonic synonymous variants, and intronic variants were removed from the analysis, while exonic non-synonymous, splice site, and loss-of-function variants were analyzed.

Classification and interpretation of sequence variants

The *APC* and *MUTYH* variants identified by Sanger sequencing and NGS analysis were classified according to the American College of Medical Genetics and Genomics and the Association of Molecular Pathology variant classification scheme^8^.

Specifically, we classified the *APC* (c.1111G>T; p.Gly371*) variant based on the following criteria:

- *APC* (c.1111G>T; p.Gly371*) is a null variant (nonsense) determining a loss of function of the *APC* protein that is known to be associated with FAP disease (OMIM # 175100) (PVS1, very strong evidence of pathogenicity);

- The *APC* (c.1111G>T; p.Gly371*) variant was found to be rare since it has never been identified in unaffected individuals and it is not listed in the global population frequency databases gnomAD^5^, 1000 genome^4^ , dbSNP^6^ and NHLBI Exome Sequencing Project^7^ (PS4, strong evidence of pathogenicity);

- The *APC* (c.1111G>T; p.Gly371*) variant was found to co-segregate with FAP disease in various affected family members included in our study and in patients described in previous studies^9^ (PP1, supporting evidence of pathogenicity).

By combining the criteria for the classification of sequence variants (1 very strong (PVS1), 1 strong (PS4), and 1 supporting (PP1)), the *APC* (c.1111G>T; p.Gly371*) variant was found to be pathogenic.

We classified the *MUTYH* (c.536A>G; p.Tyr179Cys) variant based on the following criteria:

- The *MUTYH* (c.536A>G; p.Tyr179Cys) variant has been functionally studied and has been reported to have a damaging effect on the activity of MUTYH protein^8,9^(PS3, strong evidence of pathogenicity);

- The *MUTYH* (c.536A>G; p.Tyr179Cys) variant is located in a functional domain of the MUTYH protein (DNA-binding interface that recognizes 8-oxoG:A mispairings and stabilizes protein-DNA interaction)^10^ (PM1, moderate evidence of pathogenicity);

- The *MUTYH* (c.536A>G; p.Tyr179Cys) variant has been detected in trans with other pathogenic variants in MAP patients^11^ (PM3, moderate evidence of pathogenicity);

- The *MUTYH* (c.536A>G; p.Tyr179Cys) variant was found to co-segregate with disease in various affected family members included in our study and in patients described in other studies^12–15^ (PP1, supporting evidence of pathogenicity).

- The *MUTYH* (c.536A>G; p.Tyr179Cys) variant is a missense variant and multiple lines of computational evidence support a deleterious effect on the MUTYH protein. Specifically, this variant was predicted to be deleterious by different in silico bioinformatics tools, including Sift, Polyphen, Revel, and Mutation Assessor (PP3, supporting evidence of pathogenicity).

By combining the criteria for the classification of sequence variants (1 strong (PS3), 2 moderate (PM1, PM3), and 2 supporting (PP1, PP3)), the *MUTYH* (c.1111G>T; p.Gly371*) variant was found to be pathogenic.

Literature review analysis

The literature review analysis was performed on the Human Gene Mutation Database (HGMD) Professional, a comprehensive collection of germline mutations in nuclear genes that are associated with human-inherited diseases^16^. We reviewed all the papers identified in the aforementioned database and collected clinical information (i.e., age of diagnosis, sex, number of polypoid lesions) of patients with the c.1111G>T mutation in the *APC* gene or the c.536A>G mutation in the *MUTYH* gene. Patients harboring other germline alterations or mutations or without clinical information were excluded.

1. Disciglio V, Forte G, Fasano C, et al. APC Splicing Mutations Leading to In-Frame Exon 12 or Exon 13 Skipping Are Rare Events in FAP Pathogenesis and Define the Clinical Outcome. *Genes (Basel)*. 2021;12(3):353. doi:10.3390/genes12030353

2. Groden J, Thliveris A, Samowitz W, et al. Identification and characterization of the familial adenomatous polyposis coli gene. *Cell*. 1991;66(3):589-600. doi:10.1016/0092-8674(81)90021-0

3. López-Villar I, Ayala R, Wesselink J, et al. Simplifying the detection of MUTYH mutations by high resolution melting analysis. *BMC Cancer*. 2010;10:408. doi:10.1186/1471-2407-10-408

4. 1000 Genomes Project Consortium, Auton A, Brooks LD, et al. A global reference for human genetic variation. *Nature*. 2015;526(7571):68-74. doi:10.1038/nature15393

5. Karczewski KJ, Francioli LC, Tiao G, et al. The mutational constraint spectrum quantified from variation in 141,456 humans. *Nature*. 2020;581(7809):434-443. doi:10.1038/s41586-020-2308-7

6. Smigielski EM, Sirotkin K, Ward M, Sherry ST. dbSNP: a database of single nucleotide polymorphisms. *Nucleic Acids Res*. 2000;28(1):352-355. doi:10.1093/nar/28.1.352

7. Pl A, Ap R, G W, et al. Guidelines for Large-Scale Sequence-Based Complex Trait Association Studies: Lessons Learned from the NHLBI Exome Sequencing Project. *American journal of human genetics*. 2016;99(4). doi:10.1016/j.ajhg.2016.08.012

8. Richards S, Aziz N, Bale S, et al. Standards and guidelines for the interpretation of sequence variants: a joint consensus recommendation of the American College of Medical Genetics and Genomics and the Association for Molecular Pathology. *Genet Med*. 2015;17(5):405-424. doi:10.1038/gim.2015.30

9. Stekrova J, Sulova M, Kebrdlova V, et al. Novel APC mutations in Czech and Slovak FAP families: clinical and genetic aspects. *BMC Med Genet*. 2007;8:16. doi:10.1186/1471-2350-8-16

10. Komine K, Shimodaira H, Takao M, et al. Functional Complementation Assay for 47 MUTYH Variants in a MutY-Disrupted Escherichia coli Strain. *Hum Mutat*. 2015;36(7):704-711. doi:10.1002/humu.22794

11. Wang L, Baudhuin LM, Boardman LA, et al. MYH mutations in patients with attenuated and classic polyposis and with young-onset colorectal cancer without polyps. *Gastroenterology*. 2004;127(1):9-16. doi:10.1053/j.gastro.2004.03.070

12. Colebatch A, Hitchins M, Williams R, Meagher A, Hawkins NJ, Ward RL. The role of MYH and microsatellite instability in the development of sporadic colorectal cancer. *Br J Cancer*. 2006;95(9):1239-1243. doi:10.1038/sj.bjc.6603421

13. Balaguer F, Castellví-Bel S, Castells A, et al. Identification of MYH mutation carriers in colorectal cancer: a multicenter, case-control, population-based study. *Clin Gastroenterol Hepatol*. 2007;5(3):379-387. doi:10.1016/j.cgh.2006.12.025

14. Croitoru ME, Cleary SP, Di Nicola N, et al. Association between biallelic and monoallelic germline MYH gene mutations and colorectal cancer risk. *J Natl Cancer Inst*. 2004;96(21):1631-1634. doi:10.1093/jnci/djh288

15. Fleischmann C, Peto J, Cheadle J, Shah B, Sampson J, Houlston RS. Comprehensive analysis of the contribution of germline MYH variation to early-onset colorectal cancer. *Int J Cancer*. 2004;109(4):554-558. doi:10.1002/ijc.20020

16. Stenson PD, Mort M, Ball EV, et al. The Human Gene Mutation Database: towards a comprehensive repository of inherited mutation data for medical research, genetic diagnosis and next-generation sequencing studies. *Hum Genet*. 2017;136(6):665-677. doi:10.1007/s00439-017-1779-6
