# Supplementary material for: Coinheritance of germline mutations in APC and MUTYH genes defines the clinical outcome of adenomatous polyposis syndromes
Source: Genes Dis. 2022 Dec 27;10(4):1187–9. doi: 10.1016/j.gendis.2022.11.017 (PMC10311103; doi:10.1016/j.gendis.2022.11.017)
Supplement: Multimedia component 5 [file mmc5.docx]

| Supplementary Table1. | | | | | | | | | | | |
| --- | --- | --- | --- | --- | --- | --- | --- | --- | --- | --- | --- |
| **Case** | **Gene** | **Pathogenic variant** | **Sex** | **Age at Ps diagnosis** | **N. of colorectal polyps** | **Polyp localization** | **CRC** | **Age at CRC diagnosis** | **Extracolonic manifestations** | **Reference** |  |
| I:7 | MUTYH | Y179C/*wt* | M | 55 | 10 | COLON | YES | 66 |  | this paper |  |
| I:6 | MUTYH | Y179C/*wt* | F |  | No polyps |  | NO | 40 | Gastric cancer | this paper |  |
| II:10 | MUTYH | Y179C/*wt* | F | 46 | Multiple | COLON | NO |  |  | this paper |  |
| 15311 | MUTYH | Y179C/*wt* | F |  | No polyps |  | YES | 77 |  | Colebatch et al. 2006 |  |
| 10355 | MUTYH | Y179C/*wt* | M |  | No polyps |  | YES | 76 |  | Colebatch et al. 2006 |  |
| 14838 | MUTYH | Y179C/*wt* | F |  | No polyps |  | YES | 64 |  | Colebatch et al. 2006 |  |
| 28037 | MUTYH | Y179C/*wt* | M |  | No polyps |  | YES | 65 |  | Balaguer et al. 2007 |  |
| 13028 | MUTYH | Y179C/*wt* | M |  | No polyps |  | YES | 64 |  | Balaguer et al. 2007 |  |
| 20005 | MUTYH | Y179C/*wt* | F |  | No polyps |  | YES | 69 |  | Balaguer et al. 2007 |  |
| 24057 | MUTYH | Y179C/*wt* | M |  | No polyps |  | YES | 79 |  | Balaguer et al. 2007 |  |
| C544 | MUTYH | Y179C/*wt* | M | 72 | 6 |  | YES | 72 |  | Enholm et al. 2003 |  |
| 6 | MUTYH | Y179C/*wt* | M |  | No polyps |  | YES | 59 |  | Wang et al. 2004 |  |
| 7 | MUTYH | Y179C/*wt* | M | 59 | >150 |  | YES | 59 |  | Wang et al. 2004 |  |
| 8 | MUTYH | Y179C/*wt* | M |  | No polyps |  | YES | 51 |  | Wang et al. 2004 |  |
| 9 | MUTYH | Y179C/*wt* | M |  | No polyps |  | YES | 71 |  | Wang et al. 2004 |  |
| 16 | MUTYH | Y179C/*wt* | F |  | No polyps |  | YES | 48 |  | Wang et al. 2004 |  |
| 34 | MUTYH | Y179C/*wt* | M | 63 | 100-500 |  | YES | 63 |  | Wang et al. 2004 |  |
| 36 | MUTYH | Y179C/*wt* | M | 50 | Multiple |  | NO |  |  | Wang et al. 2004 |  |
| 13 | MUTYH | Y179C/*wt* | M | 57 | 10 |  | YES | 57 |  | Croitoru et al. 2004 |  |
| 14 | MUTYH | Y179C/*wt* | M | 46 | Multiple |  | YES | 46 |  | Croitoru et al. 2004 |  |
| 15 | MUTYH | Y179C/*wt* | M |  | No polyps |  | YES | 66 |  | Croitoru et al. 2004 |  |
| 16 | MUTYH | Y179C/*wt* | M |  | No polyps |  | YES | 58 |  | Croitoru et al. 2004 |  |
| 17 | MUTYH | Y179C/*wt* | F |  | No polyps |  | YES | 65 |  | Croitoru et al. 2004 |  |
| 18 | MUTYH | Y179C/*wt* | M |  | No polyps |  | YES | 66 |  | Croitoru et al. 2004 |  |
| 19 | MUTYH | Y179C/*wt* | F | 35 | 2 |  | YES | 35 |  | Croitoru et al. 2004 |  |
| 20 | MUTYH | Y179C/*wt* | M |  | No polyps |  | YES | 56 |  | Croitoru et al. 2004 |  |
| C471 | MUTYH | Y179C/*wt* | M |  | No polyps |  | YES | 53 |  | Fleischmann et al. 2004 |  |
| 1803 | MUTYH | Y179C/*wt* | M |  | No polyps |  | YES | 79 |  | Martin-Morales et al. 2018 |  |
| II:12 | APC/  MUTYH | G371**/wt*  Y179C/*wt* | F | 43 | >30 | COLON | NO |  |  | this paper |  |
| II:13 | APC/  MUTYH | G371**/wt;*  Y179C/*wt* | M | 42 | 53 | COLON | NO |  |  | this paper |  |
| I:8 | APC | G371**/wt* | F | 63 | 5 | COLON | YES | 64 |  | this paper |  |
| I:9 | APC | G371**/wt* | M |  |  | COLON | YES | 54 |  | this paper |  |
| I:11 | APC | G371**/wt* | M | 52 | 21 | COLON | YES | 55 |  | this paper |  |
| II:11 | APC | G371**/wt* | F | 43 | >100 | COLON | YES | 43 |  | this paper |  |
| 220 | APC | G371**/wt* | F | 36 | <100 |  | NO |  |  | Sterakova et al. 2007 |  |
| 220-father | APC | G371**/wt* | M |  |  |  | YES | 50 |  | Sterakova et al. 2007 |  |
| 220-sister | APC | G371**/wt* | F | 40 | <100 |  | NO |  |  | Sterakova et al. 2007 |  |

CRC = colorectal cancer; Ps = gastrointestinal polyps.

*References*

1. Balaguer F, Castellví-Bel S, Castells A, et al. Identification of MYH mutation carriers in colorectal cancer: a multicenter, case-control, population-based study. *Clin Gastroenterol Hepatol*. 2007;5(3):379-387. doi:10.1016/j.cgh.2006.12.025

2. Colebatch A, Hitchins M, Williams R, Meagher A, Hawkins NJ, Ward RL. The role of MYH and microsatellite instability in the development of sporadic colorectal cancer. *Br J Cancer*. 2006;95(9):1239-1243. doi:10.1038/sj.bjc.6603421

3. Enomoto M, Konishi M, Iwama T, Utsunomiya J, Sugihara KI, Miyaki M. The relationship between frequencies of extracolonic manifestations and the position of APC germline mutation in patients with familial adenomatous polyposis. *Jpn J Clin Oncol*. 2000;30(2):82-88. doi:10.1093/jjco/hyd017

4. Wang L, Baudhuin LM, Boardman LA, et al. MYH mutations in patients with attenuated and classic polyposis and with young-onset colorectal cancer without polyps. *Gastroenterology*. 2004;127(1):9-16. doi:10.1053/j.gastro.2004.03.070

5. Croitoru ME, Cleary SP, Di Nicola N, et al. Association between biallelic and monoallelic germline MYH gene mutations and colorectal cancer risk. *J Natl Cancer Inst*. 2004;96(21):1631-1634. doi:10.1093/jnci/djh288

6. Fleischmann C, Peto J, Cheadle J, Shah B, Sampson J, Houlston RS. Comprehensive analysis of the contribution of germline MYH variation to early-onset colorectal cancer. *Int J Cancer*. 2004;109(4):554-558. doi:10.1002/ijc.20020

7. Martin-Morales L, Rofes P, Diaz-Rubio E, et al. Novel genetic mutations detected by multigene panel are associated with hereditary colorectal cancer predisposition. *PLoS One*. 2018;13(9):e0203885. doi:10.1371/journal.pone.0203885

8. Stekrova J, Sulova M, Kebrdlova V, et al. Novel APC mutations in Czech and Slovak FAP families: clinical and genetic aspects. *BMC Med Genet*. 2007;8:16. doi:10.1186/1471-2350-8-16
